# Effects of selected Palestinian plants on the in vitro exsheathment of the third stage larvae of gastrointestinal nematodes

**DOI:** 10.1186/s12917-017-1237-7

**Published:** 2017-11-03

**Authors:** Rana Majed Jamous, Mohammed Saleem Ali-Shtayeh, Salam Yousef Abu-Zaitoun, Alex Markovics, Hassan Azaizeh

**Affiliations:** 1 Biodiversity& Environmental Research Center –BERC, Til, Nablus, Palestine; 2 Kimron Veterinary Institute, Ministry of Agriculture and Rural Development, Jerusalem, Israel; 3 The Institute of Applied Research, The Galilee Society, P.O.B. 437, 20200 Shefa-Amr, Israel; 4 Tel Hai College, Department of Environmental Science, 2208 Upper Galilee, Israel

**Keywords:** Anthelmintics, Exsheathment inhibition assay (LEIA), Medicinal plants, Polyphenolic compounds, Tannins, Palestinian flora

## Abstract

**Background:**

Gastrointestinal parasites are one of the main restrictions to small ruminant production. Their pathological importance is primarily related to the major production losses, in quantity or quality, induced by the direct action of worms. Control of these parasites is based exclusively on the frequent use of anthelmintic drugs. However, the resistance to anthelmintics in worm populations after commercialisation of chemical drugs is now widespread. Therefore, there is a need to find new natural resources to ensure sustainable and effective treatment and control of these parasites. The aim of this study was to evaluate the anthelmintic activity, as minimum inhibitory concentration (*IC*_*50*_ mg/mL), of different plant extracts using larval exsheathment inhibition assay using a two-species but steady population of parasitic nematodes (ca. 20% *Teladorsagia circumcinta* and 80% *Trichostrongylus colubriformis*).

**Results:**

The study showed that the ethanolic extracts of 22 out of the 48 plant extracts, obtained from 46 plant species, have an inhibitory effect >50% (at concentrations of 100 mg/mL) on the third stage larvae (L3) of the nematodes exhibited the strongest inhibition activity (94%) with *IC*_*50*_ of 0.02 mg/mL, where other members of the Rhamnaceae family have shown to possess strong anthelmintic activity (70–89%).

**Conclusions:**

Plant extracts are potential rich resources of anthelmintics to combat helminthic diseases. Our results suggest that extracts from *Rhamnus elaternus, Epilobium hirsutum, Leucaena leucocephala* and *Rhamnus palaestinus* have promising anthelmintic activity, with potential applications in animal therapeutics and feed.

## Background

Infections of the gastrointestinal tract of ruminants with parasitic nematodes represent a major pathology associated with outdoor production of sheep and goats worldwide [1–4]. Small ruminant are infected with the same prominent gastrointestinal parasites, which induce similar pathological symptoms and economic consequences [5]. Gastrointestinal nematodes are one of the main restrictions to sheep and goats production and may cause more than 20% loss for marketable products [6]. Their pathological importance is primarily related to the major production losses, in quantity or quality, induced by the direct action of worms. However, other studies suggested that the host’s immune-mediated response in grazing meat-breed lambs infected with *T. colubriformis* is the major component of production loss [7].

Control of gastrointestinal parasites is based exclusively on the frequent use of anthelmintic drugs. However, this apparently exclusive reliance on synthetic molecules is not sustainable and currently faces several limitations. The first one is the rapid development of resistance to anthelmintics in worm populations after commercialisation of chemical drugs and the occurrence, in some regions, of multi-resistant strains strongly challenges the control of these parasites with drugs nowadays [8–11]. Second, is the increased concern of consumers over drug residues in meat and milk products, and a potential risk for environmental contamination [12]. In the attempt to reduce the use of chemicals due to the concern about human health and environmental toxicology, new and safer food control approaches such as the use of natural compounds are nowadays being developed [13–15].

In vivo and in vitro studies have shown that plants containing secondary metabolites, such as tannins, sesquiterpene lactones and flavone glycosides, are a promising option for use in integrated nematode control in farm production systems [16–18]. Tannin-rich plant extracts have shown in vitro to prevent eggs hatching of nematodes, and hinder development, motility, and exsheathment of larvae [19–21]. In addition, feeding of goats and sheep with tanniferous plants has led to reduction of nematode egg excretion and worm burden [22–25]. Some differences in activity of the same tannin-rich resource might be depending on the tested parasite species.

Natural compounds from plants provide a remarkable opportunity in the search for new, effective and safe anthelmintic products [26–28]. In Palestine, medicinal plants have been reported to play a vital role in the treatment of many disease conditions in humans [29–32]. A recent study has revealed that around 140 medicinal plant species are still in use in traditional medicine in Palestine for the treatment of several livestock diseases including gastro-intestinal infections (causing diarrhea, colic, flatulence, constipation, digestive system, and anthelmintic) [33]. This research also showed the gastrointestinal disorders is the disease group in the study area that scored the highest informant consensus factor (ICF) value (0.90), followed by urinary, and reproductive disorders (0.89) [33]. However, these plants have not been experimentally evaluated for their anthelmintic activity to justify their use in the treatment of such infections. The current study was therefore aimed at: collection of potential local plant species from Palestine, extraction of the bioactive ingredients, and determining the total phenolic and tannin contents; evaluation of the anthelmintic activity of different plant extracts using larval exsheathment inhibition assay (LEIA), and determination of the minimum inhibitory activity (*IC*_*50*_) of the most active plants extracts.

## Methods

### Preparation of plant extracts for exsheathment tests.

Areal parts of 46 plant species belonging to 26 families were collected from open fields during the period from June 2015 to February 2016 from Palestine, where plant species were identified by Prof. M. S. Ali-Shtayeh from the Biodiversity and Environmental Research Center, BERC, Til Village, Nablus (Table 1). Voucher specimens are deposited in the Herbarium of BERC. Plant parts were dried at 50 °C for 24 h, then ten grams from each dried material was grinded and incubated separately with 100 mL of 70% ethanol at 35 °C for 3 h. The extracts were then filtered and dried using rotary evaporator at 50 °C to remove the ethanol followed by freeze drying. The powdered extracts were stored at − 20 °C for further analysis [18]. Extraction yield was calculated as g extract per g dried matter (DM) of plant leaves.

**Table 1:** List of local flora analyzed for their exsheathment inhibition activity (LEIA) of the third stage larvae (L3) of a mixture of nematodes species

Scientific name (Family) (Voucher specimen)	English Name	Plant part	Total phenolic content (GAE-mg/g)	Tannins (GAE-mg/g)	% Exsh. inhibition (60 min)	IC50 (mg/mL)
*Rhamnus alaternus L.* (Rhamnaceae) (BERC-BX-C-0124)	Italian Buckthorn	Leaves	68.1	46.35	94	0.02
*Rhamnus palaestinus* Boiss. (Rhamnaceae) (BERC-BX-C-0150)	Palestine Buckthorn	Leaves	126.85	86.6	89	0.22
*Epilobium hirsutum* L. (Onagraceae) (BERC-BX-C-0250)	Great Willowherb	Leaves	234.58	98.68	84	0.03
*Pistacia palaestina* Boiss. (Anacardiaceae) (BERC-BX-C-0010)	Palestinian Pistachio, Terebinth	Leaves	121.63	58.93	84	0.50
*Laurus nobilis* L. (Lauraceae) (BERC-BX-C-0061)	Laurel, Sweet Bay	Leaves	96.98	84.58	79	0.37
*Ziziphus sativa* Gaetn. (Rhamnaceae) (BERC-BX-C-0231)	Jujube	Leaves	136.75	86.75	76	2.09
*Leucaena leucocephala* (Lam.) (Fabaceae) (BERC-BX-C-0264)	Leucaena	Leaves	136.85	69.45	74	0.10
*Ziziphus spina-christi* (L.) Desf. (Rhamnaceae) (BERC-BX-C-0186)	Christ’s Thorn Jujube	Leaves	131.23	80.8	70	1.78
*Myrtus communis* L. (Myrtaceae) (BERC-BX-C-0051)	Common Myrtle	Leaves	143.93	72.43	69	0.73
*Juglans regia* L. (Juglandaceae) (BERC-BX-C-0230)	Wallnut	Leaves	90.58	42.55	68	1.23
*Sarcopoterium spinosum* (L.) Spach. (Rosaceae) (BERC-BX-C-0084)	Shruppy Barnet	Areal Parts	215.93	103.48	65	0.37
*Dittrichia viscosa* (L.) Greuter (Asteraceae) (BERC-BX-C-0032)	Inula	Areal Parts	162.8	73.4	63	0.40
*Rhus coriaria* L. (Anacardiaceae) (BERC-BX-C-0037)	Sicilian Sumach	Leaves	256.78	19.68	62	0.30
*Olea europaea* L. (Oleaceae) (BERC-BX-C-0086)	Olives	Leaves	80.43	47.6	60	2.02
*Acacia saligna* (Labill.) Wendl. f. (Mimosaceae) (BERC-BX-C-0042)	Blue-Leafed Wattle	Leaves	154	74.9	55	
*Carthamus tenuis* (Boiss. & Blanche) Bornm. (Asteraceae) (BERC-BX-C-0582)	Safflower	Areal Parts	41.8	19.15	55	
*Satureja thymbra* L. (Lamiaceae) (BERC-BX-C-0206)	Summer Savory	Areal Parts	97.45	31.4	53	
*Quercus calliprinos* Webb (Fagaceae) (BERC-BX-C-0016)	Kermes Oak	Leaves	211.4	102.45	52	
*Cistus creticus* L. (Cistaceae) (BERC-BX-C-0157)	Cretan Rock Rose	Leaves	219.85	84.65	51	
*Nicotiana glauca* Graham (Solanaceae) (BERC-BX-C-0193)	Tobacco Tree	Leaves	31.2	11.15	50	
*Rosmarinus officinalis* L. (Lamiaceae) (BERC-BX-C-0018)	Rosemary	Areal Parts	201.85	153.15	50	
*Vitis vinifera* L. (Vitaceae) (BERC-BX-C-0111)	Grape	Leaves	81.93	51.05	50	
*Capparis spinosa* L. (Capparaceae) (BERC-BX-C-0106)	Caper Bush, Egyptian Caper	Fruits	65.15	33	49	
*Eucalyptus camaldulensis* Dehn. (Myrtaceae) (BERC-BX-C-0039)	Red River Gum	Leaves	197.23	131.05	49	
*Leucaena leucocephala* (Lam.) (Fabaceae) (BERC-BX-C-0264)	Leucaena	Fruits	39.75	14.28	49	
*Cupressus sempervirens* L. (Cupressaceae)	Cypress	Leaves	106.4	70.8	48	
*Capparis spinosa* L. (Capparaceae) (BERC-BX-C-0106)	Caper Bush, Egyptian Caper	Leaves	18.9	0.35	46	
*Solanum luteum* Mill. (Solanaceae) (BERC-BX-C-118)	Hairy Nightshade	Leaves	40.2	11.65	45	
*Ceratonia siliqua* L. (Caesalpiniaceae) (BERC-BX-C-0137)	Carob	Leaves	215.15	104.6	44	
*Heliotropium europaeum* L. (Boraginaceae) (BERC-BX-C-0161)	Common Heliotrope	Leaves	26.15	8.2	43	
*Helianthemum syriacum* (Jacq.) Dum (Cistaceae) (BERC-BX-C-0482)	Common Rock -Rose	Areal Parts	162.55	71.5	41	
*Atriplex halimus* L. (Chenopodiaceae) (BERC-BX-C-0014)	Shruppy Saltbush, Sea Orache	Leaves	19.8	9.48	39	
*Salvia fruticosa* Mill. (Lamiaceae) (BERC-BX-C-0006)	White Sage, Common Sage	Leaves	130.4	65.65	39	
*Teucrium capitatum L.* (Lamiaceae) (BERC-BX-C-0167)	Cat Thyme	Areal Parts	60.18	28.08	37	
*Pinus halepensis* Mill. (Pinaceae) (BERC-BX-C-0015)	Aleppo Pine	Leaves	81.08	53.93	36	
*Rubus sanctus* Schreb. (Rosaceae) (BERC-BX-C-0113)	Brandle, Blackberry	Leaves	154.05	72.15	34	
*Crataegus aronia* (L.) DC. (Rosaceae) (BERC-BX-C-0059)	Hawthorn, Azarole	Leaves	106.5	69.83	33	
*Cupressus arizonica* Greene (Cupressaceae)	Arizona Cypress	Leaves	126.13	68.33	32	
*Ruta chalepensis* L. (Rutaceae) (BERC-BX-C-0008)	Rue	Leaves	41.3	21.93	30	
*Teucrium creticum* L. (Lamiaceae) (BERC-BX-C-0173)	Cretan Germander	Areal Parts	52.4	19.8	29	
*Varthemia iphionoides* Boiss. & Blanche (Asteraceae) (BERC-BX-C-0135)	Common Verthemia	Areal Parts	94.3	45.58	29	
*Elaeagnus angustifolia* L. (Elaeagnaceae) (BERC-BX-C-0050)	Narrow-Leaved Oleaster	Leaves	50.23	33.95	27	
*Origanum syriacum* L. (Lamiaceae) (BERC-BX-C-0026)	Wild Thyme, Mother Of Thyme	Areal Parts	91.23	40.83	25	
*Euphorbia hierosolymitana* Boiss. (Euphorbiaceae) (BERC-BX-C-0170)	Spurge	Areal Parts	42.53	18.9	21	
*Lantana camara* L. (Verbenaceae) (BERC-BX-C-0134)	Lantana	Areal Parts	84.65	44.78	17	
*Thuja occidentalis* L. (Cupressaceae) (BERC-BX-C-0040)	Tree Of Life	Areal Parts	71.98	42.38	12	
*Retama raetam* (Forssk.) Webb (Fabaceae) (BERC-BX-C-0043)	Retama, White Broom	Areal Parts	99.48	72.7	8	
*Ficus carica* L. (Moraceae) (BERC-BX-C-0048)	Fig Tree	Leaves	35.3	13.25	7	

### Determination of total phenolic content

Total phenols in plant extracts were measured by a colorimetric assay following the Folin-Ciocalteu method [34]. In brief, 0.5 mL of each sample dissolved in methanol (1 mg/mL) was transferred to test tube and mixed with 2.5 mL of a 10 fold dilute Folin-Ciocalteu reagent and 2 mL of 7.5% sodium carbonate. The tubes were allowed to stand for 30 min at room temperature. Then the absorbance was recorded spectrometrically at 760 nm versus gallic acid as blank, and total phenolic content was expressed as mg gallic acid equivalent (GAE)/g dry weight. All samples were analyzed in three replicates.

### Determination of tannin contents

Tannin content in each plant sample was measured using the polyvinylpyrrolidone (PVP) following the method described by Makkar et al. [35]. In brief, 0.5 mL of extract dissolved in methanol (1 mg/mL) was mixed with 0.5 mL (100 mg /mL) of PVP, vortexed for few seconds, stand for 15 min at 4 °C and then centrifuged for 10 min at 3000 rpm. The non-tannin phenolics were determined in the clear supernatant in the same way as for total phenolic content. Total phenols in plant extracts were measured by the colorimetric assay following the Folin-Ciocalteu method as described above. Tannin content was calculated as the difference between total phenolic and non-tannin phenolic contents in the plant extracts.

### Larval exsheathment inhibition assays (LEIA)

We used one donor goat that was parasitized by two nematode species, *Teladorsagia circumcincta* and *Trichostrongylus colubriformis* which are considered the main species at our region and usually found in the same animal. Species were identified as described by Van Wyk et al. [36]: their proportions in the suspension were steady throughout the year: 25, 75; 25, 75; 20, 80; and 18, 82%; in winter, spring, summer, and fall, respectively. The LEIA assay was performed according to Bahuaud et al. [37]. The extracts were diluted in phosphate buffer solution (PBS), and the tested concentration was 1 mg/mL. The test was performed on three replicate samples of each plant extract as well as of samples of the PBS solution, which served as a negative control. Approximately 200 L3 larvae (150 μl) were mixed with 30 μl of PBS, and 150 μl of diluted plant extract (at a concentration of 1 mg/mL). The mixture was incubated for 3 h at 37 °C. After the incubation period, larvae were washed with PBS and centrifuged at 3000 rpm; this step was repeated three times. Artificial exsheathment was induced in vitro by adding a solution of sodium hypochlorite (2% *w*/*v*) and sodium chloride (16.5% w/v), diluted 1:300 in PBS, to the larval suspension. The kinetics of larval exsheathment process was determined by removing an aliquot containing about less than one-fifth of the larvae content at time intervals of 0, 15, 30, 45, and 60 min from exposure, and counting the number of exsheathed and ensheathed individuals under Olympus microscope CX31 (400×). Counts were averaged within each time point.

### Minimum inhibitory concentration (*IC*_*50*_)

The minimum inhibitory concentration (*IC*_*50*_), which is defined as the amount of the extract necessary to decrease the larval exsheathment by 50%, was determined by the regression between the percentage of exsheathment inhibition and concentration of the extracts (mg/mL) after 45 min incubation using Microsoft Excel Program 2010. Plant extract concentrations used were 1, 0.5, 0.25, and 0.125 (mg/mL). PBS was used as a negative control.

### Statistical analyses

The percentage of larval exsheathment in each plant extract and negative control was calculated as follow [38]:

$$ \%\mathrm{of}\  \mathrm{Exsheated}\  \mathrm{Larvae}=\left[\frac{\mathrm{Number}\  \mathrm{of}\  \mathrm{exsheated}\;\mathrm{L}3\;\mathrm{larvae}}{\begin{array}{l}\kern0.6em \mathrm{Number}\  \mathrm{of}\kern0.96em +\kern0.84em \mathrm{Number}\  \mathrm{of}\\ {}\mathrm{Ensheated}\  \mathrm{Larvae}\  \mathrm{Exsheated}\  \mathrm{larvae}\end{array}}\right]\kern0.24em \mathrm{X}\kern0.5em 100 $$

Values of semi-log slopes for the percentages of exsheathed larvae at each of the five time intervals were calculated and subjected to analysis of variance using the General Linear Model procedure of SAS (1989). For LEIA, the model comprised treatment with 50 levels (48 plant extracts × 1 extraction method, and a PBS control).

## Results

### Polyphenolic compounds and tannins

**Fig. 1:**
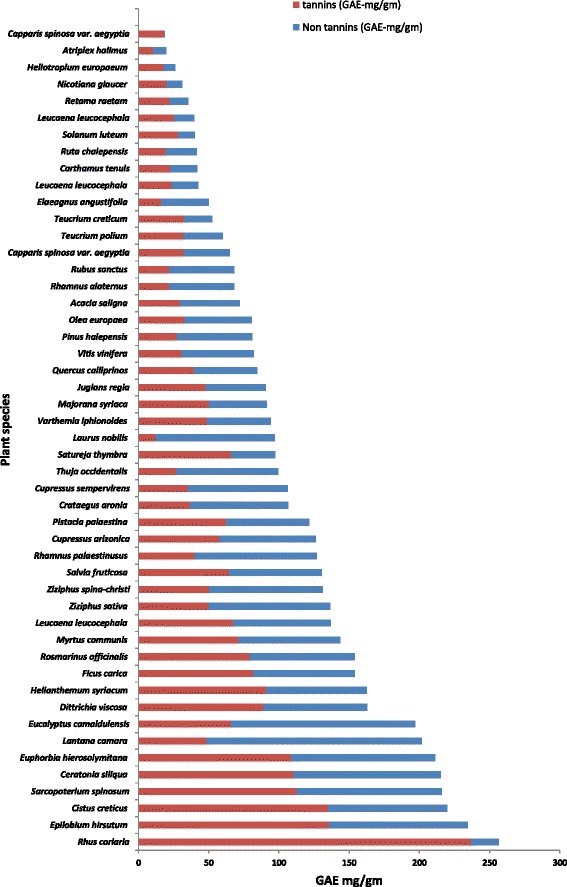
Total tannins and non-tannin contents of the tested plant extracts

Extraction yields ranged between 13 and 37% depending on the plant species. The polyphenols concentration expressed as gallic acid equivalent are shown in Table 1 and Fig. 1. Total polyphenols levels in plant extracts were ranged from 18.9 mg/g GAE in *Capparis spinosa* leaves to 256.8 mg/g GAE in *Rhus coriaria* (Fig. 1, Table 1). On the other hand tannin levels were ranged from 0.35 mg/g GAE in *C. spinosa* leaves to 153.2 mg/g GAE in *Rosmarinus officinalis* (Fig. 1, Table 1). It is worth noting that, although *Capparis spinosa* possess very low levels of total phenols and tannins it showed moderate exsheathment inhibition activity (46%).

### Larval exsheathment inhibition assay (LEIA)

The exsheathment of the infective third stage larvae (L3) of the mixed-species nematodes in the control groups was similar in all assays with more than 90% of exsheathment obtained after 60 min of incubation. In the tested plant extracts, after 3 h of contact with each plant extract, the percentage of exsheathment inhibition activity, obtained after 60 min, ranged between 7 and 94% where *Rhamnus elatarnus, Rhamnus. palaestinus, Pistacia palaestina,* and *Epilobium hirsutum* showed the highest percentage of exsheathment inhibition activity of 94, 89, 84, and 84%, respectively (Table 1).

**Fig. 2:**
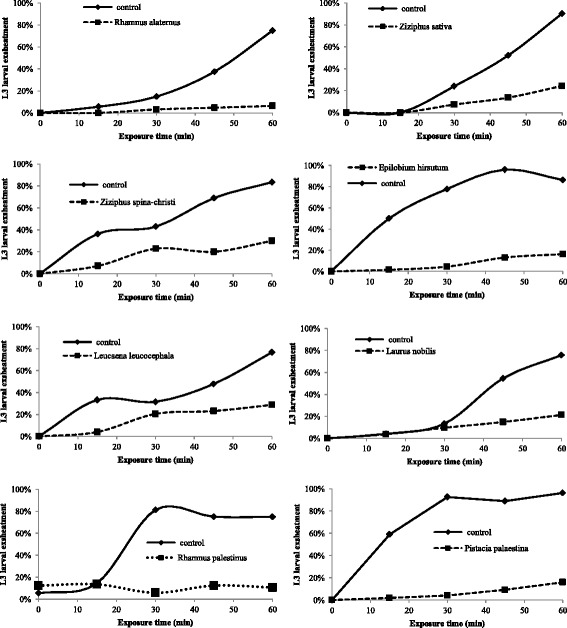
Minimum inhibitory concentration (*IC*_*50*_) of 8 plant extracts which exhibited >70% exsheathment inhibition activity

Out of the 48 plant extracts tested, 22 extracts showed exsheathment inhibitory activity of ≥50%, where 17 exhibited LEIA of ≥30% - < 50%, and 9 plant extracts showed <30% LEIA. The minimum inhibitory concentration *IC*_*50*_ was calculated for the 14 plant extracts that showed ≥60% exsheathment inhibition activity (Table 1). *Rhamnus alaternus* extracts which possess relatively moderate levels of total phenols (68.1 mg/g) and tannins (46.35 mg/g), have shown the highest LEIA (94%) and lowest *IC*_*50*_ (0.02 mg/mL, Table 1 and Fig. 2). On the other hand, some plant extracts showed low exsheathment inhibition activity despite having relatively high total phenolic content. Examples of those were: *Crataegus aronia*, (106.5 mg/g, 33% LEIA), *Cupressus arizonica* (126.13, 32), *Salvia fruticosa* (130.4, 39), and *Rubus sanctus* (154.1, 34). However, no significant correlation was detected in this study between total phenolic or tannin contents and the % of exsheathment inhibition activity.

## Discussion

The eggs of parasitic strongyles are excreted in the animal faeces, hatch under suitable environmental conditions especially on pasture, producing two non-parasitical larval stages followed by a third-stage infective larvae (L3) that is ensheathed, i.e., retaining the shed cuticle from the previous moult for protection. L3 exsheathment is therefore a critical process in the life cycle, being a transitionary step from the free-living to the parasitic stages where its exsheathment inhibition is considered as a crucial stage of its control [39]. Using a two-species but steady population of parasitic nematodes (ca. 20% *Teladorsagia circumcinta* and 80% *Trichostrongylus colubriformis*) was used in different works since both are found in the same animal [40].

In our results the Rhamnaceae family was represented by 4 species (*Rhamnus alaternus*, *Rh. palaestinus*, *Ziziphus spina-christi*, *Z. sativa*). The four species were among the 10 plant extracts with highest LEIA (Table 1). Different parts of the plants (leaves, aerial parts, seeds, fruits, and roots) belonging to the Rhamnaceae family are traditionally used in traditional medicine for the treatment of many illnesses such as inflammation, fever, insomnia, relieve pain, anthelmintic, weakness, hepatic disorders, obesity, urinary problems, muscle spasm and viral infections [41–44]. The Biological activity studies on these species revealed different medicinal features including antioxidant [45, 46], antimicrobial [46–48], anti-diabetic [49] and antifungal [50] activities. In addition, some of these species are grazed by animals including goats and sheep where polyphenol content may affect larval reproduction.

The Rhamnaceae species are known to produce a variety of characteristic secondary compounds including triterpenes, cyclopeptides alkaloids, benzylisoquinoline alkaloids (BIAs), and flavonoids [51]. Several studies have mentioned the importance of some phytochemicals like alkaloids, glycosides, terpenoids, tannins and flavonoids for showing anthelmintic activity of plant extracts [52, 53]. In this study we assume that the polyphenols and flavonoids were responsible for the anthelmintic activity of the Rhamnaceae plants which needs further investigation. We acknowledge that the value of the Folin-Ciocalteu to determine total phenolics is controversial. The extraction of phenolic compounds from plant materials is influenced by different factors including: chemical structure, mineral nutrients, physiological processes, the extraction method, the solvent of extraction, the size of the particles forming samples, the time of sampling, extraction time, the conditions of storage as well as the presence of interfering compounds. Inorganic substances may also interact with Folin-Ciocalteu reagent giving varying results; in addition the structural features of phenolics are another parameter that should be considered when this assay is applied. However, it is the most straightforward measurement of phenolics and enables comparison of published data. In addition, we used PVP method in order to test total tannins out of the total phenols despite we know there are other methods to precipitate tannins from plant extracts such as alkaline ethanol [54].

Other plants which have shown LEIA >70% included *Epilobium hirsutum* (84), *Pistacia palaestina* (84), *Laurus nobilis* (79), and the leaves of *Leucaena leucocephala* (74)*.*

*Epilobium hirsutum* has shown to possess a strong antioxidant and anticandidal activity [55], the plant contains polyphenols including steroids (especially β-sitosterol and its esters), tannins (gallic, protocatechuic, ellagic and p-coumaric acids) and flavonoids (in particular myricetin, isomyricetin, quercetin, and 5-quercetin-3-O-β-d-glucuronide) [56–58]. The isolated β-sitosterol from the methanolic extract of rhizomes of *Hedychium spicatum* has shown to possess a strong anthelmintic activity [59], thus the presence of β-sitosterol and tannins in *E. hirsutum* might explain its strong LEIA in this study.

*Pistacia palaestina* is used in traditional Palestinian herbal medicine for weight loss, and for the treatment of diabetes, urinary tract infections, kidney gallstone, and neuropathic pain [42]. In this study the plant exhibited strong LEIA (84%) with *IC*_*50*_ of 50 μg /mL. A strong LEIA was also reported to *P. atlantica* of the same genus and family with very low *IC*_*50*_value of 3.5 μg /mL [18].

*Laurus nobilis* leaves are used as a spice for cooking purposes, the plant is used traditionally for the treatment of gastric diseases [42, 60]. The plant leaves have been reported to possess wound healing, neuroprotective, antioxidant, antiulcerogenic, anticonvulsant, antimutagenic, antiviral, anticholinergic, antibacterial, and antifungal activities [61]. The anthelmintic activity of the plant can be associated with the presence of various sesquiterpene lactones which have been reported to be present in the plant [62]. Williams et al., [63], have attributed the anthelmintic activity of *Cichorium intybus* to its sesquiterpene lactones composition.

*Leucaena leucocephala* is often used as feed for livestock [64, 65]. Leucaena possesses anthelmintic properties against both *Haemonchus contortus* [66] and *Trichostrongylus colubriformis* [67]. In our study the leaves of *Leucaena* have shown strong LIEA with *IC*_*50*_ of 100 μg/mL. The anthelmintic activity of the plant might be attributed to the protease inhibitor activity of the plant extracts [68]. Another study conducted in 2015 [69] performed a bio-guided fractionation of an aqueous extract of *Leucaena leucocephala* leaves using the egg hatch assay (EHA) to identify the phytochemicals present in fresh leaves with anthelmitic activity. The isolation procedures used led to the identification of an active fraction mainly composed of quercetin (82.21%) and caffeic acid (13.42%) which inhibited the egg hatching of *Cooperia* spp. by 90.49 ± 2.8% of (*P* < 0.05).

Tannins are the most studied compounds for anthelmintic activity, however, some researchers have shown that the ethanolic extracts of *Pistacia lentiscus* still possess some anthelmintic activity remains after all tannins are bound by polyethyleneglycol [70]. In this study, some plants possess low levels of total phenols and tannin content, e.g., *Nicotiana glauca*, and *Capporis spinosa*, however these plants exhibited a moderate LEIA. *Nicotiana glauca* is an herbaceous plant with high toxicity due to its primary alkaloid anabasine content [71]. Although *N. glauca* possesses a low total phenolic and tannin contents the plant exhibited 50% EIA, the anthelmintic activity of the plant might be attributed to its toxicity. However, the aqueous and methanolic extracts of plants of the same genus (*N. tabacum*) have shown to possess anthelmintic activity when administrated orally for sheep naturally infected with mixed species of gastrointestinal nematodes [72].

*Capparis spinosa* fruits and leaves have been tested in this study for their LEIA, the tannin content ranged between 0.35 mg/g in leaves to 33 mg/g in fruits, with LEIA of 46, and 49%, respectively. The anthelmintic activity of ethanolic extract of root bark of *C. spinosa* was evaluated against earthworm, and the activity was found to be dose dependent [73]. Traditional medicine plays a vital role in the development of new drugs; a lot of plant-originated drugs in clinical medicine today were derived from traditional medicine [74–76]. Fifteen of the plant species analyzed in this study for their LEIA were mentioned to be used traditionally in ethnoveterinary medicine in Palestine for the treatment of several animal ailments [33]. Nine of these plants were reported to be used for the treatment of gastrointestinal diseases including diarrhea, and anthelmintic (Table 2). Our results support these ethnoveterinary studies and using these plants as grazing pasture or feed additives might contribute to a sustainable control of these nematodes in small ruminants.

**Table 2:** Ethnoveterinary uses of plants used in Traditional Arabic Palestinian Herbal medicine (TAPHM) [33]

Plant	Ethnoveterinary uses
*Ceratonia siliqua*	Nourishment, anthelmintic, scabies, eye inflammation, cleaning uterus, delay in giving birth, diarrhea
*Crataegus aronia*	Urine retention, constipation, inflammation, coccidia, pregnancy poisoning
*Cupressus sempervirens*	Diarrhea
*Dittrichia viscosa*	Expectorant, appetizer, flatulence, postpartum inflammation
*Eucalyptus camaldulensis*	Fever & malaria, flatulence, bees anaesthetization, diarrhea, scabies, inflammation
*Ficus carica*	Ulcers, insects bites, constipation, nourishment
*Myrtus communis*	Diarrhea, ulcers
*Olea europaea*	Eye diseases, bone fractures, anthelmintic, bruises, nourishment, injuries, scabies, colic, flatulence, diarrhea
*Origanum syriacum*	Flatulence, inflammation, Bites, cold, appetizer
*Rosmarinus officinalis*	Animal fertility, arthritis, inflammation, constipation
*Salvia fruticosa*	Appetizer, cleaning the uterus, colic, flatulence, poisoning, cold, diarrhea, satiety
*Solanum luteum*	Dimples, scabies, injuries
*Teucrium capitatum*	Diarrhea, colic, bleeding, scabies, flatulence
*Varthemia iphionoides*	Colic, scabies diarrhea, fever, flatulence, pregnancy poisoning, udder infections
*Ziziphus spina-christi*	Diarrhea, nourishment, anthelmintic, inflammation, carbuncles

## Conclusion

Our results suggest natural resources that possess strong larval exsheathment inhibition activity with potential applications in animal therapeutics and feed. In our study the calculated *IC*_*50*_ values for the most active extracts of *Rhamnus alaternus,* and *Epilobium hirsutum* were 20, and 30 μg /mL, respectively. This is a strong indication of the high anthelmintic potential of these plants. Future studies are needed for screening in-depth phytochemical, clinical and possible studies on molecular mechanism of action. At the same time efforts should be made to normalize the plant extracts with potent anthelmintic activity and formulate best alternative herbal products to either substitute or supplement man-made drugs which are presently in use.

## Abbreviations

DM: Dry matter
EIA: Exsheathment inhibition activity
GAE: Gallic acid equivalents
*IC*_*50*_: Minimum inhibitory concentration
LEIA: Larval exsheathment inhibition assays
PBS: Phosphate buffered saline
PVP: Polyvinylpyrrolidone
